# Effects of *Vigna angularis* extract and its active compound hemiphloin against atopic dermatitis-like skin inflammation

**DOI:** 10.1016/j.heliyon.2023.e12994

**Published:** 2023-01-20

**Authors:** Seon Gyeong Bak, Hyung Jin Lim, Eun Jae Park, Yeong Seon Won, Seung Woong Lee, Soyoung Lee, Sang-Ik Park, Seung Jae Lee, Mun-Chual Rho

**Affiliations:** a Functional Biomaterial Research Center, Korea Research Institute of Bioscience and Biotechnology (KRIBB), Jeongeup, 56212, South Korea; b College of Veterinary Medicine, Chonnam National University, Gwangju, 61186, South Korea; c Applied Biological Engineering, KRIBB School of Biotechnology, University of Science and Technology, Daejeon, 34113, South Korea

**Keywords:** Atopic dermatitis, Skin inflammation, *Vigna angularis*, Hemiphloin

## Abstract

*Vigna angularis* is an edible crop and herbal medicine that is known to have antipyretic, anti-inflammatory, and anti-edema effects. Many studies have been conducted on the 95% ethanol extract of *V. angularis*, but there is little research on the 70% ethanol extract and hemiphloin, which is a new indicator component of the 70% ethanol extract of *V. angularis*. To investigate the *in vitro* anti-atopic effect and verify the mechanism action of 70% ethanol extract of *V. angularis* (VAE), TNF-α/IFN-γ-induced HaCaT keratinocytes were used. The VAE treatment alleviated TNF-α/IFN-γ-induced IL-1β, IL-6, IL-8, CCL17/TARC, and CCL22/MDC gene expressions and productions. VAE also inhibited the phosphorylation of MAPKs, including p38, ERK, JNK, STAT1, and NF-κB in TNF-α/IFN-γ-induced HaCaT cells. 2,4-dinitochlorobenzene (DNCB)-induced skin inflammation mice model, and HaCaT keratinocytes were used. In the DNCB-induced mouse model, VAE treatment alleviated ear thicknesses and IgE levels. Furthermore, VAE decreased IL-1β, IL-6, IL-8, CCL17/TARC, and CCL22/MDC gene expressions of DNCB-applied ear tissue. Additionally, we investigated the anti-atopic and anti-inflammatory effects of hemiphloin using TNF-α/IFN-γ-induced HaCaT keratinocytes and LPS-induced J774 macrophages. Treatment hemiphloin decreased gene expressions and productions of IL-1β, IL-6, IL-8, CCL17/TARC, and CCL22/MDC in TNF-α/IFN-γ-induced HaCaT cells. The phosphorylations of p38, ERK, STAT1, and NF-κB were inhibited by hemiphloin in TNF-α/IFN-γ-induced HaCaT cells. Finally, hemiphloin showed anti-inflammatory activities in LPS-induced J774 cells. It decreased LPS-induced NO productions and iNOS and COX-2 expressions. Treatment of hemiphloin also inhibited LPS-induced TNF-α, IL-1β, and IL-6 gene expressions. These results suggest that VAE is an anti-inflammatory agent for inflammatory skin diseases and that hemiphloin could be a therapeutic candidate for inflammatory skin diseases.

## 1 Introduction

The skin is the front-line organ in the protection against external factors and is composed of several cells such as keratinocytes, epithelial cells, macrophages, and mast cells [1]. Among these cells, macrophages play a central role in innate immunity and an important role in skin defense. Activation of skin resident macrophages by external stimuli results in the production of many related mediators, such as cytokines and chemokines, which act as mediators and induce inflammation of the epidermis [2,3]. In atopic dermatitis-like inflammatory skin diseases, cytokines and mediators exacerbate chronic inflammatory conditions. Additionally, inflammatory chemokines, a group of cytokines, recruit inflammatory cells to lesioned skin causing immune cell infiltration at the inflammatory site. In particular, thymus and activation-regulated chemokines (CCL17/TARC) and macrophage-derived chemokines (CCL22/MDC), which are regulated upon inflammatory activation, are produced in keratinocytes and are considered therapeutic targets for the treatment of inflammatory skin diseases [4,5]. Therefore, mediating the expression of atopic dermatitis-related cytokines and chemokines is considered a therapeutic strategy because these factors act as important regulators of the subsequent inflammatory response. *Vigna angularis* has been used as a folk remedy in Northeast Asia since ancient times. Previous studies have reported that it has excellent anti-oxidant (K. J. [6], anti-bacterial [7], and protective effects against aging and neurodegeneration [8]. Recently, 95% ethanol extract of *V. angularis* was reported to exert effect against osteoporosis and osteoarthritis [9], alleviate glucose intolerance in diabetic rat liver [10], and ameliorate mast cell-mediated allergic inflammation [11]. Although a lot of research has been conducted on the 95% ethanol extract of *V. angularis*, research on the 70% ethanol extract is limited. Moreover, we isolated hemiphloin as a new indicator component of 70% ethanol extract of *V. angularis.* Hemiphloin is a C-glucosyl-flavonoid [12], which is found on kino of *Eucalyptus hemiphloia* F. Muell [12,13], Leaves of *Betula platyphylla* var. *latifolia* (M.-W. [14], and bark of Schoepfia chinensis [15]. Nevertheless, research on hemophloin in the treatment of the disease is unclear. Therefore, we investigated the effect of the 70% ethanol extract of *V. angularis* on atopic dermatitis.

## 2 Materials and methods

### 2.1 Preparation of *V. angularis* and isolated hemiphloin

The dried seeds of *V. angularis* were extracted with 70% EtOH at 70 °C (6 h) and filtered (No. 10, 600 mm, Hyundai Micro Co., Seoul, South Korea). Then, 1.6 kg of extract was obtained by concentration of filtrates under reduced pressure. The extract was suspended in distilled water, and the aqueous layer was partitioned with *n*-hexane, EtOAc, and BuOH. The *n*-hexane layer (1.1 kg) was subjected to silica gel column elution with CHCl_3_: MeOH (100:1 → 0:100, v/v) to obtain 16 fractions (PAH1 ∼ PAH16). Fraction 1 (PAH1) and fraction 12 (PAH12) were subjected to MPLC [Teledyne Isco-U.S., Combi Flash Rf, C18; mobile phase: MeOH: H_2_O (1:9 → 0:10, v/v)]. Hemiphloin (3.3 mg) was obtained from fraction PAH12H (174.0 mg) by preparative HPLC (Phenomenex Luna C_18_ column 250 × 21.2 mm, 5 μm), using isocratic elution with 17% CH_3_CN in H_2_O.

#### 2.1.1 Hemiphloin

Yellow powder; Chemical formula C_21_H_22_O_10_; ESI-MS *m/z* 435.4 [M+H]^+^; ^1^H NMR (600 MHz, MeOD-*d*_*4*_) *δ*_H_ 7.31 (2H, d, *J* = 8.4 Hz, H-2ʹ, H-6ʹ), 6.81 (2H, d, *J* = 8.4 Hz, H-3ʹ, H-5ʹ), 5.96 (1H, s, H-5), 5.36 (1H, dd, *J* = 12.6, 3.0 Hz, H-2), 4.78 (1H, d, *J* = 10.2 Hz, H-1ʹʹ), 4.12 (1H, m, H-2ʹʹ), 3.85 (1H, m, H-6ʹʹα), 3.71 (1H, m, H-6ʹʹβ), 3.43 (2H, m, H-3ʹʹ, H-5ʹʹ), 3.37 (1H, m, H-4ʹʹ), 3.13 (1H, d, *J* = 17.4, 6.6 Hz, H-3α), 2.75 (1H, d, *J* = 17.4, 3.0 Hz, H-3β); ^13^C NMR (150 MHz, MeOD-*d*_*4*_) *δ*_C_ 161.8 (C-5), 128.5 (C-2ʹ, 6ʹ), 115.4 (C-3ʹ, 5ʹ), 81.6 (C-5ʹʹ), 79.2 (C-3ʹʹ), 78.4 (C-2), 73.1 (C-1ʹʹ), 71.0 (C-4ʹʹ), 70.5 (C-2ʹʹ), 61.7 (C-6ʹʹ), 42.1 (C-3).

### 2.2 Generation of 2,4-dinitochlorobenzene (DNCB)-induced atopic dermatitis-like lesions

Six-week-old female BALB/c mice were obtained from Samtako (Osan, Korea). A total of 25 mice were divided into five groups (n = 5): the vehicle phosphate-buffered saline (PBS), DNCB vehicle (PBS), DNCB and VAE (100 and 300 mg/kg), and DNCB and dexamethasone (DX, 5 mg/kg) groups. For sensitization, 20 μL of DNCB was applied once each ear (face side and back side of ear, once) in first week. Then, both ears of BALB/c mouse were challenged with DNCB (1%, 20 μL/ear, once) for 3 weeks. VAE (100 and 300 mg/kg) or DX (5 mg/kg) was orally administered by gavage for ﬁve consecutive days per week at the time of the DNCB challenge. Mice were provided with ad libitum access to food and water. The room was maintained with a 12 h light-dark cycle (lights on at 07:00 and lights off at 19:00) and controlled temperature (21 ± 2 °C). Mice were handle in accordance with the guidelines established by the Public Health Service Policy on the Humane Care and Use of Laboratory Animals and the experimental protocols were approved by the Institutional Animal Care and Use Committee of Korea Research Institute of Bioscience and Biotechnology (permission number #KRIBB-AEC-21259).

### 2.3 Histological assay

Mouse ear tissues were fixed in 4% (w/v) paraformaldehyde in PBS, (pH 7.4). The fixed samples were embedded in paraffin and sectioned. Then, the specimen were stained with hematoxylin and eosin (H&E).

### 2.4 Cell culture

The human keratinocyte cell line, HaCaT (ATCC, Rockville, MD, USA) was maintained in Dulbecco's modiﬁed Eagle's medium (DMEM), supplemented with 10% heat-inactivated fetal bovine serum (FBS) and 1% penicillin-streptomycin. The murine macrophage cell line J774 was maintained in DMEM, supplemented with 10% non-heated FBS and 1% penicillin–streptomycin. The cells were incubated at 37 °C in 90–95% humidity and 5% CO_2_.

### 2.5 Cell viability

Cell viability was determined by the 3-(4, 5-dimethylthiazol-2-yl)-2, 5-diphenyl tetrazolium bromide (MTT) assay. HaCaT and J774 cells were seeded at a density of 3 × 10^4^ cells/well in 200 μL culture medium in 96-well plates and incubated for 24 h at 37 °C. The cells were treated with VAE or hemiphloin at several concentrations; 10, 30, 60, and 100 μg/mL VAE, and 1, 10, and 100 μM hemiphloin. After 24 h, the cells were treated with MTT solution for 3 h and supernatant was removed. Then, the crystallized formazan was dissolved in DMSO and the absorbance at 540 nm was measured using a microplate reader.

### 2.6 Measurement of nitric oxide (NO) levels

J774 cells were pretreated with hemiphloin or DX for 1 h and stimulated with 100 ng/mL lipopolysaccharide (LPS) for 24 h. After the incubation, nitrite levels in medium were determined using a NO Plus Detection Kit (iNtRON Bio, Seongnam, Korea). Briefly, 50 μL of supernatant was mixed with an equal volume of NO Plus Detection kit reagent. The absorbance at 540 nm was measured using a microplate reader.

### 2.7 Real-time quantitative polymerase chain reaction (qPCR)

TRIzol regent was used for isolation of RNA. The complementary DNA (cDNA) was synthesized using a PrimeScript 1st Strand cDNA Synthesis Kit (Takara Bio Inc., Shiga, Japan). Real-time qPCR was performed using a Bio-Rad T100 thermal cycler (Bio-Rad, Hercules, CA, USA) with TaqMan probes and TaqMan Real-Time PCR Master mix (Applied Biosystems, Foster City, CA, USA) according to the manufacturer's protocol. The gene expressions were normalized by 18s rRNA and GAPDH, as an endogenous control. The information of TaqMan probe was provided in Table 1.

**Table 1:** Information of TaqMan probe.

Name	Probe
Interleukin (IL)-1β	Hs01555410_m1
	Mm00434228_m1
IL-6	Hs00174131_m1
	Mm00446190_m1
IL-8	Hs00174103_m1
CCL17	Hs00171074_m1
	Mm01244826_g1
CCL22	Hs01574247_m1
	Mm00436439_m1

### 2.8 Enzyme-linked immunosorbent assay (ELISA)

The level of cytokine (IL-1β, IL-6, and IL-8) and chemokine (CCL17/TARC, CCL22/MDC) in cell conditioned medium, and serum IgE were determined using ELISA kits (BD Biosciences, San Diego, CA, USA; R&D Systems, Minneapolis, MN; Thermo Fisher Scientific, Waltham, Massachusetts, USA, respectively) according to the manufacturer's instructions. The absorbance was measured at 450 nm using a microplate reader.

### 2.9 Western blotting

Detail protein extraction and Western blot procedures were described in the previous study [16]. In brief, HaCaT cells (1 × 10^6^ cells/well in 6-well plates) were pretreated with VAE, hemiphloin, or cyclosporine for 1 h. For 30 min, stimulated with TNF-α (50 ng/mL)/IFN-γ (50 ng/mL). J774 cells (1 × 10^6^ cells/well in 6-well plates) were pretreated with hemiphloin or DX for 1 h and then stimulated with LPS for 24 h. The cells were lysed with 100 μL of cell lysis buﬀer (Cell Signaling Technology, Danvers, MA, USA) and centrifuged at 13,000 rcf for 10 min at 4 °C. The supernatants were harvested and quantification were performed by DC protein assay kit (Bio-Rad, Contra Costa County, CA, USA). Equally quantified protein lysate was subjected to electrophoresis on an SDS-PAGE gel and then transferred onto a polyvinylidene ﬂuoride (PVDF) membrane. After blocking with 5% bovine serum albumin in Tris-buffered saline for 1 h, the membrane was incubated with primary antibody against the target protein, 3 times washed, and subsequently incubated with horseradish peroxidase-conjugated IgG secondary antibody. The bands were developed using a West-Queen RTS Western Blot Detection Kit (iNtRON Bio, Seongnam, Korea). Antibodies purchased from Cell Signaling Technology and Santa Cruz were used (Table 2.).

**Table 2:** Information of antibodies.

Name		Dilution ratio	Cat. No.	Company
phosphorylation(p)-p38	rabbit monoclonal	1:1000	#4511S	Cell Signaling Technology
p38	rabbit monoclonal	1:1000	#8690S	
p-ERK	rabbit monoclonal	1:1000	#9101S	
ERK	rabbit monoclonal	1:1000	#4348S	
β-Actin	rabbit monoclonal	1:1000	#4967S	
p-NF-κB	rabbit polyclonal	1:1000	#3033S	
COX-2	rabbit monoclonal	1:1000	#12282S	
iNOS	rabbit monoclonal	1:1000	#13120S	
p-JNK	mouse polyclonal	1:1000	#sc-6254	Santa Cruz
JNK	mouse polyclonal	1:1000	#sc-7345	
p-STAT-1	mouse polyclonal	1:1000	#sc-8394	

### 2.10 Statistical analysis

The data are presented as the mean ± SD of three for *in* vitro or five for *in vivo* individual experiments. Statistical analysis was performed using Prism 5 software (GraphPad Software, San Diego, CA, USA) and statistical significance was determined by one-way ANOVA followed by Tukey's multiple comparisons test.

## 3 Results

### 3.1 Effect of VAE on viability and proinflammatory gene expressions in HaCaT cells

**Fig. 1:**
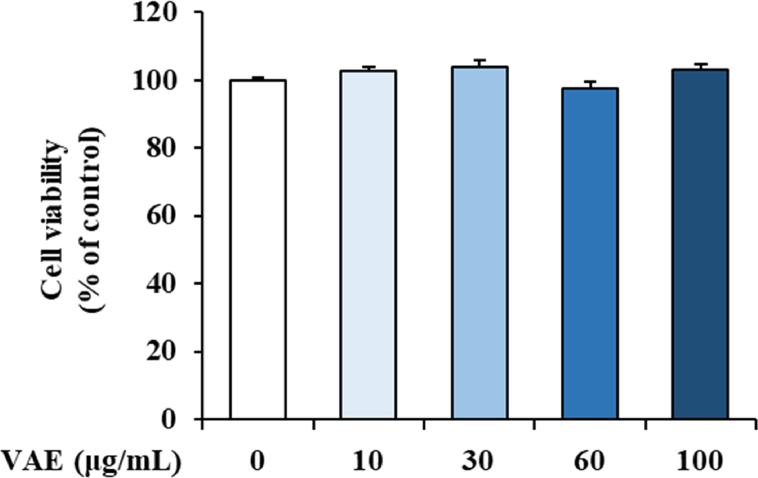
Cell viability of VAE on HaCaT cells. Cell viability of various treatment concentration of VAE in HaCaT cells. HaCaT cells were treated with VAE at the indicated concentrations for 24 h.

**Fig. 2:**
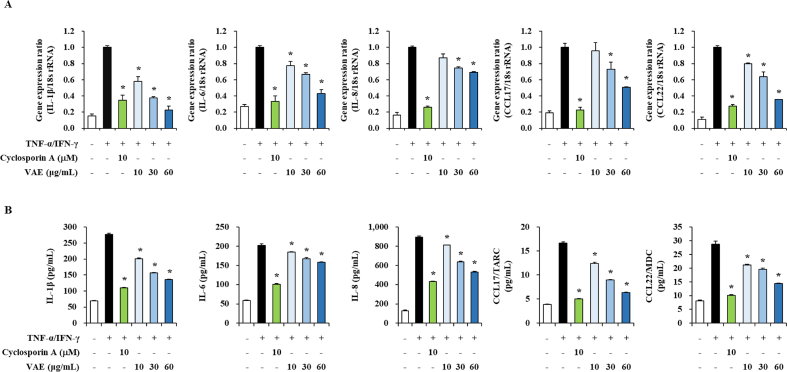
Effect of VAE on proinflammatory factor gene expression and production in TNF-α/IFN-γ-stimulated HaCaT cells. (A) The level of proinflammatory cytokines and chemokines were measured by real-time qPCR. (B) The levels of secreted proinflammatory cytokines and chemokines were measured by ELISA. *p < 0.05 compared with the TNF-α/IFN-γ-stimulated group.

HaCaT cells, which are human keratinocytes, were treated with various concentrations to examine the effect of VAE on cell viability. After 24 h, cell viability was measured by MTT assay (Fig. 1). VAE at concentrations of 10, 30, 60, and 100 μg/mL did not have any significant toxic effects. Thus, in all experiments, we used 10, 30, and 60 μg/mL VAE. We evaluated the anti-inflammatory effect of VAE at the level of cellular on HaCaT cells, and analyzed the expression of proinflammatory cytokines and chemokines under TNF-α/IFN-γ-induced inflammatory conditions in HaCaT human keratinocytes. HaCaT cells were pretreated with VAE (10, 30, and 60 μg/mL), and then stimulated with 50 ng/mL TNF-α/IFN-γ, respectively, 1 h later. In HaCaT cells stimulated with TNF-α/IFN-γ, high gene expression of proinflammatory factors such as IL-1β, IL-6, IL-8, CCL17, and CCL22 was observed. However, pretreatment with VAE inhibited the expression of proinflammatory genes and proteins stimulated by TNF-α/IFN-γ in a concentration-dependent manner (Fig. 2A and B). Our data showed that VAE suppressed inflammatory gene and protein expression in keratinocytes.

### 3.2 Effect of VAE on HaCaT cells intracellular signaling

**Fig. 3:**
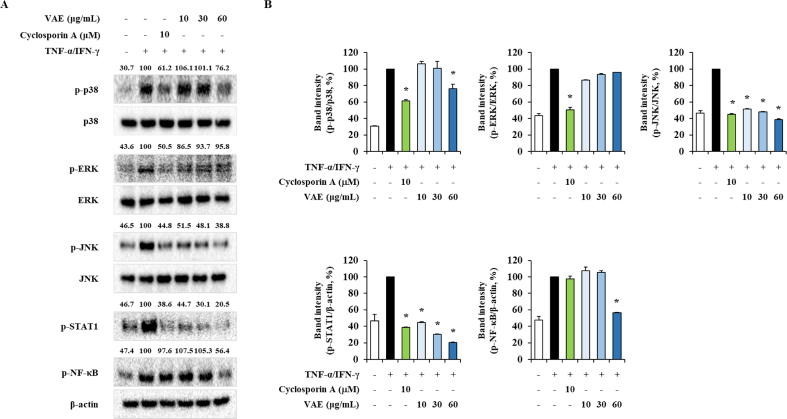
Effect of VAE on intracellular signal transduction in TNF-α/IFN-γ stimulated HaCaT cells. (A) HaCaT cells were pretreated with 10, 30 or 60 μg/mL VAE for 1 h before stimulation with TNF-α/IFN-γ for 30 min, and total protein was extracted using Thermo Fisher total protein extraction reagent. (B) The band intensity were normalized by TNF-α/IFN-γ stimulated group using ImageJ software. *p < 0.05 compared with TNF-α/IFN-γ-stimulated group.

To demonstrate the mechanism underlying the anti-inflammatory effect of VAE, the intracellular signaling pathway stimulated by TNF-α/IFN-γ was evaluated. MAPKs, including p38, EKR, and JNK, have been found to exert a wide spectrum of bioactivities at the cellular level, such as effect on the cell cycle and, metabolism and inflammation activity [17,18]. Phosphorylation of STAT-1 has been reported to induce atopic dermatitis by including a Th2 responses [19]. In addition, NF-κB is a transcription factor that plays and important role in the expression of inflammatory gene [18,19]. Therefore, MAPKs, STAT-1 and NF-κB may be therapeutic targets for the atopic inflammatory responses. HaCaT cells stimulated with TNF-α/IFN-γ showed high phosphorylation of signaling molecules such as p38, ERK, JNK, STAT-1 and NF-κB. However, it was confirmed that 60 μg/mL of VAE inhibited phosphorylated p38, JNK, and NF-κB due to TNF-α/IFN-γ-stimulation. (Fig. 3). β-Actin was used as a loading control. Our data indicated that VAE downregulates the expression of inflammatory-associated signaling molecules, to exert anti-inflammatory effects.

### 3.3 Effect of VAE on DNCB-induced atopic dermatitis

**Fig. 4:**
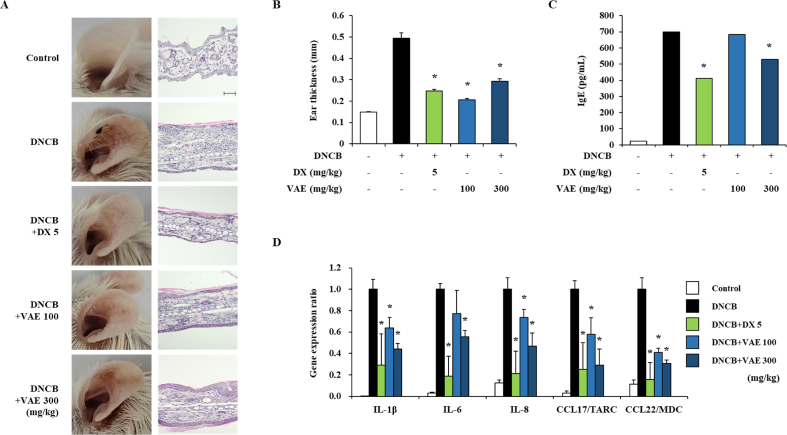
Effect of VAE on skin lesions and IgE levels in DNCB-induced atopic dermatitis mouse model. (A) Skin lesions on the ears of mice with DNCB-induced atopic dermatitis. Both ears each mouse were treated with 20 μL of 1% DNCB for 3 weeks. VAE (100 and 300 mg/kg) or DX (5 mg/kg) was orally administered by gavage for ﬁve consecutive days per week at the time of DNCB treatment. The image shows ear skin 24 h after the last treatment with DNCB. The sections of ear tissue were evaluated at original magnification of 200 × , scale bar = 100 μm. (B) The ear thickness of DNCB-treated mice was measured 24 h after the last DNCB treatment using a digital ear thickness gauge. (C) Serum IgE levels were measured by ELISA. Serum was collected immediately after mice with DNCB-induced atopic dermatitis were sacrificed. (D) Total RNA was extracted from ear tissues, and the levels of proinflammatory cytokines such as IL-1β, IL-6 and IL-8, and chemokines such as CCL17 and CCL22 were measured by real-time qPCR. *p < 0.05 compared with the DNCB-treated group.

We evaluated the effects of VAE using and atopic-like animal model. The DNCB has been used to induce atopic skin lesions and is a powerful agent. [20]; H. N [21]. As shown in Fig. 4A, in the DNCB-treated group, atopic skin lesions were significantly aggravated compared to the vehicle group. It was confirmed that the thickness of the ear visually confirmed was increased by DNCB, and histopathological analysis showed an increase in the thickness of the epidermis and dermis through H&E staining. On the other hand, oral administration of VAE reduced DNCB-induced atopic skin lesions. (Fig. 4A and B). In atopic dermatitis, IgE is and immunoglobulin involved in the Th2 response [22]. The DNCB-treatment group showed significantly increased serum IgE levels. However, oral administration of VAE decreased serum IgE levels (Fig. 4C). According to previous studies, epithelial cells and keratinocytes damaged by atopic dermatitis produce proinflammatory cytokines (such as TNF-α, IL-1β, IL-6, and IL-8) and chemokines (such as CCL17/TARC, CCL22/MDC) [18]. Therefore, we confirmed the inhibitory effect of proinflammatory cytokines and chemokines expressed by atopic dermatitis in ear tissue by oral administration of VAE. The DNCB-treatment group showed marked elevation of the levels of pro-inflammatory cytokines and chemokines, such as IL-1β, IL-6, IL-8, CCL17, and CCL22. However, oral administration of VAE decreased the expression levels of these genes (Fig. 4D). Our data indicated that VAE attenuates atopic dermatitis-related skin lesions.

### 3.4 Effect of hemiphloin on HaCaT cell viability and proinflammatory gene expressions in HaCaT cells

**Fig. 5:**
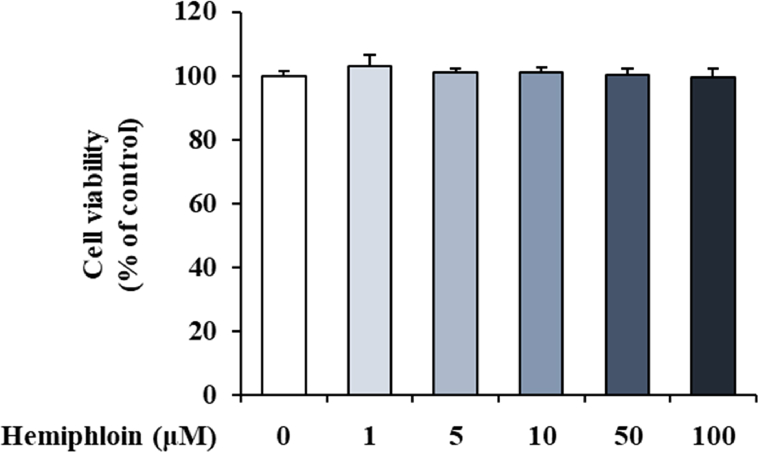
Cell viability of Hemiphloin on HaCaT cells. Cell viability of various treatment concentration of hemiphloin in HaCaT cells.

**Fig. 6:**
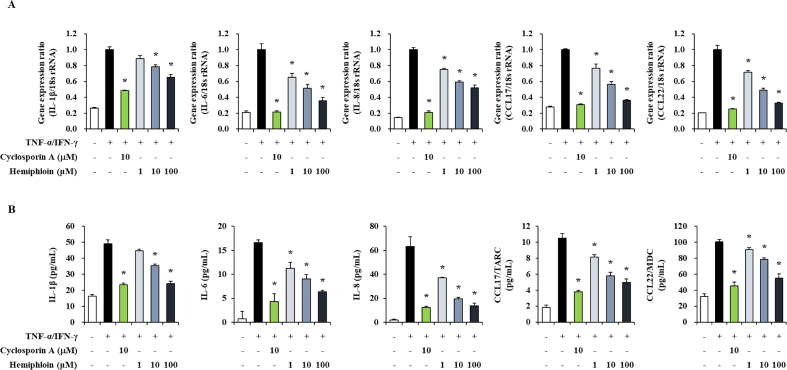
Effect of hemiphloin on pro-inflammatory gene expression and production levels in TNF-α/IFN-γ stimulated HaCaT cells. (A) The level of pro-inflammatory cytokines and chemokines were measured by real-time qPCR. (B) The level of secreted pro-inflammatory cytokines and chemokines were measured by ELISA. *p < 0.05 compared with TNF-α/IFN-γ-stimulated group.

The effects of hemiphloin have not been reported. Thus, we conducted an experiment to confirm the activity of hemiphloin in HaCaT cells. To explore the effect of hemiphloin on the viability of HaCaT cells, we first treated cells with the indicated concentrations of hemiphloin and then measured cell viability after 24 h using the MTT assay. Hemiphloin at concentrations of 1, 10, and 100 μM did not have any significant toxic effects. (Fig. 5). HaCaT cells were pretreated with various concentrations of hemiphloin (1, 10, and 100 μM) for 1 h before stimulation with TNF-α/IFN-γ. TNF-α/IFN-γ-stimulated HaCaT cells showed increases in the gene expression and production of inflammatory factors, such as proinflammatory cytokines and chemokines. However, pretreatment with hemiphloin inhibited the expression and production of proinflammatory factor genes induced by TNF-α/IFN-γ in a concentration-dependent manner (Fig. 6A and B). In addition, the highest concentration of hemiphloin showed an inhibitory effect similar to that of cyclosporine A, a positive control drug. Our data suggested that hemiphloin strongly regulates expression of inflammatory genes in keratinocytes.

### 3.5 Effect of hemiphloin on HaCaT cells intracellular signaling

**Fig. 7:**
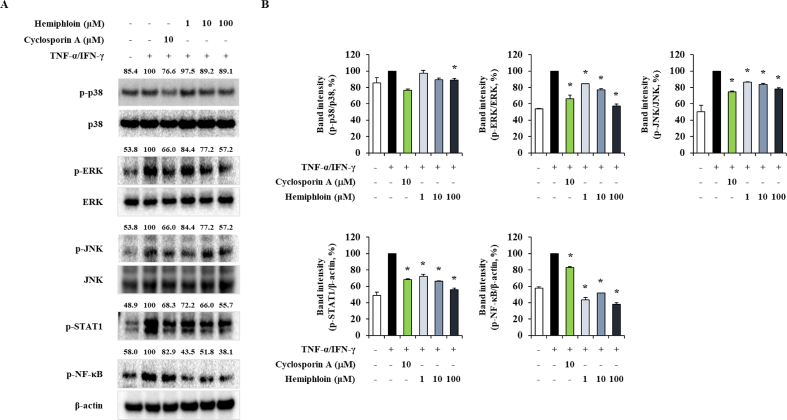
Effect of hemiphloin on intracellular signal transduction in TNF-α/IFN-γ stimulated HaCaT cells. (A) HaCaT cells were pretreated with 1, 10 or 100 μg/mL hemiphloin for 1 h before stimulation, with TNF-α/IFN-γ for 30 min, and total protein was extracted using Thermo Fisher total protein extraction reagent. (B) The band intensity were normalized by TNF-α/IFN-γ stimulated group using ImageJ software. *p < 0.05 compared with TNF-α/IFN-γ-stimulated group.

To explore the mechanism of action of hemiphloin, the intracellular signaling pathway stimulated by TNF-α/IFN-γ was evaluated. It was shown that phosphorylation of signaling molecules was activated by TNF-α/IFN-γ in HaCaT cells. However, pretreatment with hemiphloin suppressed the TNF-α/IFN-γ-induced phosphorylation of signaling molecules (Fig. 7). Furthermore, it was confirmed that the phosphorylation of MAPKs, STAT-1, and NF-κB was inhibited in a concentration-dependent manner through treatment with hemiploin, and significantly inhibited at high concentration. Our data indicated that hemihloin potently modulates atopic skin lesions by regulating intracellular signaling in keratinocytes.

### 3.6 Effect of hemiphloin on NO and COX-2 production and expression of proinflammatory genes in J774 cells

**Fig. 8:**
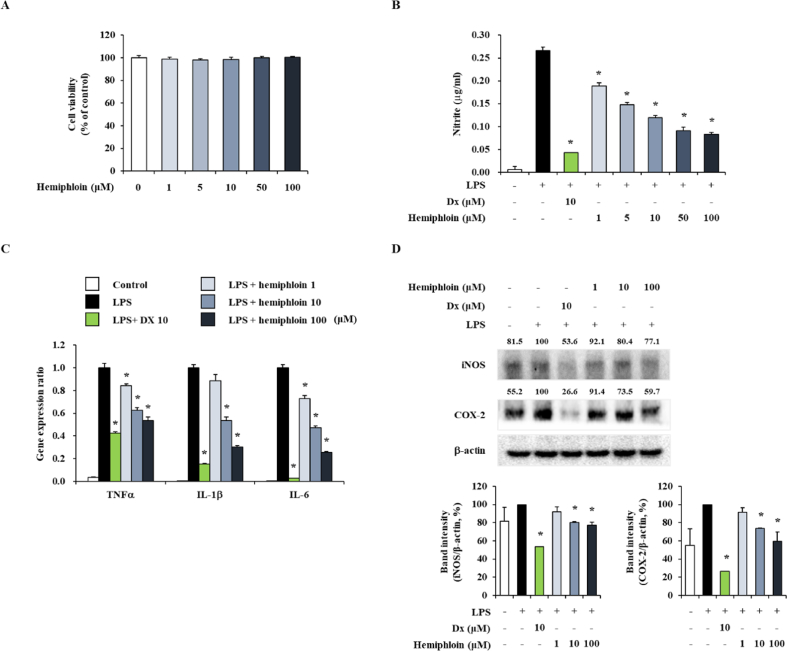
Effect of hemiphloin on proinflammatory factor gene expression and production in LPS stimulated J774 cells. (A) Cell viability of various treatment concentration of hemiphloin in J774 cells. J774 cells were treated with hemiphloin at the indicated concentrations for 24 h. (B) J774 cells were pre-treated with 1, 10 or 100 μg/mL of hemiphloin for 1 h before the stimulation. Then, J774 cells were stimulated with LPS for 24 h, and RNA of cells were extracted using Trizol reagent. (C) The level of proinflammatory cytokines and chemokines were measured by real-time qPCR. (D) The level of secreted were measured by Western blot. The band intensity were normalized by LPS stimulated group. *p < 0.05 compared with LPS-stimulated group.

To confirmed the efficacy of hemiphloin in marophage cells other than keratinocyte cells using J774 cells. As shown in HaCaT cells, hemiphloin was not cytotoxic to J774 cells. (Fig. 8A). Additionally, to determine the inhibitory effects of hemiphloin on LPS-induced NO production, we used an NO Plus Detection Kit. J774 cells were pretreated with various concentrations of hemiphloin (1, 10, and 100 μM) for 1 h before stimulation with LPS. The supernatants from J774 cells stimulated with LPS were recovered to measure NO production levels. As shown in Fig. 8B, compared with control cells, LPS-stimulated J774 cells showed increases in the production of NO, and cells treated with various concentrations of hemiphloin showed a dose-dependent decrease in the increased NO production level. DX (10 μM), a well-known anti-inflammatory drug was used as a positive control. Because hemiphloin attenuated the production of NO, we examined whether this effect was related to decrease in the transcript levels of the TNF-α, IL-1β, and IL-6 genes, and iNOS, and COX-2 production. J774 cells were pretreated with different concentrations of hemiphloin for 1 h and then stimulated with 100 ng/mL of LPS, for 24 h. As a result of real-time PCR analysis, the mRNA expression of TNF-α, IL-1β, and IL-6 were significantly upregulated by LPS stimulation compared to the vehicle group. On the other hand, it was confirmed that inflammatory cytokines induced by LPS were reduced when cells were pretreated with various concentrations of hemiphloin. (Fig. 8C). In addition, the levels of iNOS and COX-2 were increased by LPS treatment, whereas cells treated with the indicated concentrations of hemiphloin showed a dose-dependent decrease in LPS-induced iNOS and COX-2 production (Fig. 8D). These results indicated that hemiphloin inhibits the production of inflammatory response genes induced by LPS in J774 cells.

## 4 Discussion

Atopy is one of the most common forms of chronic eczema and affects thousands of people worldwide. Atopic dermatitis is an allergic reactions in the skin barrier. The lesion of acute atopic dermatitis is confined to hives, rashes, and itching. However, in the chronic state occurs to severe skin damage, resulting in a breakdown of the patient's skin immune system and psychological distress. Therefore, the development of therapeutic agents with safe and high efficacy was required. We demonstrated the pharmacological effects of 70% ethanol extract of *V. angularis* on DNCB-induced skin inflammation using *in vitro* and *in vivo* models.Our results showed that VAE inhibited TNF-α/IFN-γ-stimulated production of cytokines in keratinocytes. In addition, the level of MAPKs, STAT-1, and NF- κB was alleviated by VAE. In the inflammatory environment, damaged keratinocytes release the many inflammatory cytokines such as IL-1β, IL-6, and IL-8, which leads to pathogenesis of skin inflammation [5]. These cytokine expressions were requested by activation of signaling molecules such as MAPKs, STAT-1, and NF-κB, which play essential roles in several inflammatory responses, including atopic dermatitis [23]. Therefore, VAE can effectively suppress the increase in the thickness of the epidermis and dermis and the expression of inflammatory cytokines by suppressing the inflammation-related signaling pathway. *V. angularis* contains several compounds, and these compounds have been reported to exert various pharmacological effects [23, 24, 25, 26]. However, hemiphloin, a new indicator component, is isolated from 70% EtOH extract of *V. angularis*. In addition, we investigated the anti-inflammatory effects of hemiphloin in TNF-α/IFN-γ-stimulated HaCaT keratinocytes. Hemiphloin exerted inhibitory effects on cytokine production and the phosphorylation of MAPKs, STAT-1, and NF-κB in TNF-α/IFN-γ-stimulated HaCaT keratinocytes. Our results indicated that the anti-inflammatory effects of VAE are due to the inhibition of cytokine expression through suppressing the cell signaling pathway. Inflammatory mediators and cytokines exacerbate the inflammatory response to skin lesions in skin diseases such as atopic dermatitis [27]. In addition, chemokines are small, heparin-binding proteins that have chemotactic activity for selective cell populations [5]. CCL chemokines are sub-family of chemokines, play essential roles in allergic inflammation because they potently attract eosinophils, basophils, monocytes and Th2 cells [28]. Clinical studies have reported that increases in CCL17/TARC and CCL22/MDC levels in patient samples are associated with worsening of atopic lesions [29,30]. In the context of inflammatory skin diseases, CCL17/TARC and CCL22/MDC production is increased epidermal keratinocytes upon stimulation with TNF-α/IFN-γ, resulting infiltration of immune cells into the lesion area [5]. Our date showed that VAE inhibited CCL17 and CCL22 levels in TNF-α/IFN-γ-stimulated HaCaT keratinocytes and DNCB-induced atopic dermatitis animal models. Moreover, hemiphloin also suppressed the expression of chemokines in TNF-α/IFN-γ-stimulated HaCaT keratinocytes. These results indicated that anti-atopic effects of VAE is due to alleviation of cytokines as well as chemokines expression.A mentioned above, damage to the skin layer is induced by secreted inflammatory cytokines, leading to inflammatory responses such as the expression of inflammatory cytokines and chemokines [31]. In atopy, macrophages activated by foreign substances secrete proinflammatory cytokines such as TNF-α and IFN-γ [32]. Morevoer, activated macrophage releases NO through up-regulation of intracellular enzyme activities such as iNOS and COX-2. Previous studies showed that iNOS-derived NO levels aggravates pathogenesis of atopic dermatitis lesions in animal model [33]. COX-2, a major pro-inflammatory mediator, was also found to be induced by LPS, resulting in the secretion of PGD_2_ and PGE_2_, which play a role in the allergic and inflammatory responses [34]. Therefore, level of iNOS and COX-2 can be indicated to evaluate the severity of atopic dermatitis, and the interaction between macrophages and keratinocytes may be a target for atopy treatment. Hemiphloin significantly reduced the production of COX-2, iNOS and cytokines in LPS-stimulated J774 cells. Our results suggest that hemiphloin suppressed activation of macrophages and keratinocytes.

## 5 Conclusion

In this study VAE significantly inhibited chemokine and cytokine production induced by TNF-α/IFN-γ and DNCB *in vitro* and *in vivo*. Hemiphloin which was isolated from *V. angularis* extract, also significantly decreased chemokine and cytokine production by TNF-α/IFN-γ *in vitro*. Hemiphloin reduced the levels of these mediators of atopic dermatitis-like inflammation, such as NO, COX-2, iNOS, and cytokines in LPS-stimulated J774 cells. These results indicate that VAE and hemiphloin can act as preventive and therapeutic agents for inflammation-related skin diseases.

## Author contribution statement

Seon Gyeong Ba, Hyung Jin Lim: Conceived and designed the experiments; Wrote the paper.

Eun Jae Park, Yeong Seon Won, Sang Ik Park: Performed the experiments; Analyzed and interpreted the data.

Seung Woong Lee, Soyoung Lee, Mun-Chual Rho: Contributed reagents, materials, analysis tools or data.

Seung-Jae Lee: Conceived and designed the experiments; Analyzed and interpreted the data; Wrote the paper.

## Funding statement

This work was supported by the Korea Institute for Advancement of Technology (P0010383), Ministry of Oceans and Fisheries and the Korea Research Institute of Bioscience and Biotechnology (KGM5242221).

## Data availability statement

Data included in article/supplementary material/referenced in article.

## Declaration of interest’s statement

The authors declare no competing interests.
