# Supplementary material for: Effects of Vigna angularis extract and its active compound hemiphloin against atopic dermatitis-like skin inflammation
Source: Heliyon. 2023 Jan 20;9(2):e12994. doi: 10.1016/j.heliyon.2023.e12994 (PMC9922827; doi:10.1016/j.heliyon.2023.e12994)
Supplement: Multimedia component 1 [file mmc1.pdf]

Western blotting images

Figure 3A

p-p38	p38
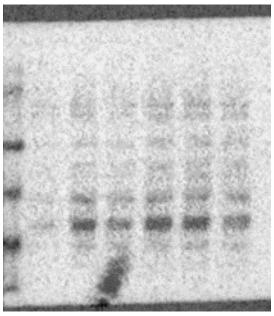	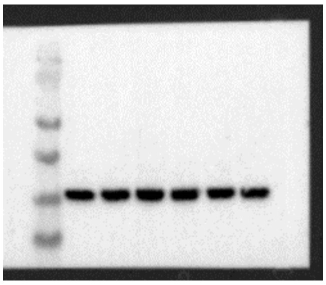
**p-ERK**	**ERK**
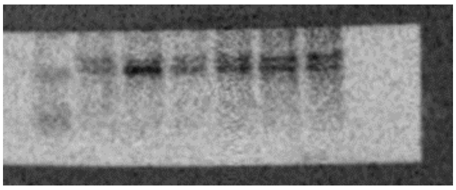	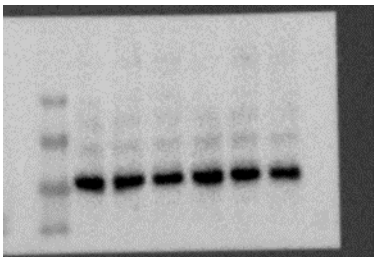
**p-JNK**	**JNK**
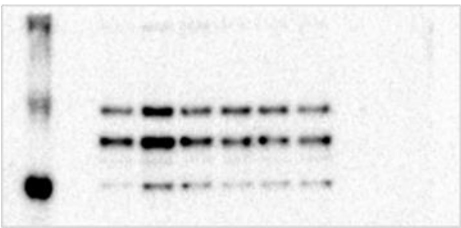	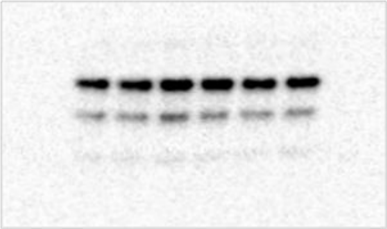
**p-STAT1**	**p-NF-κB**
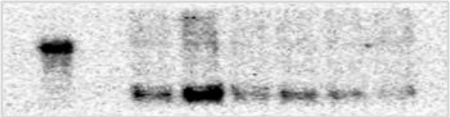	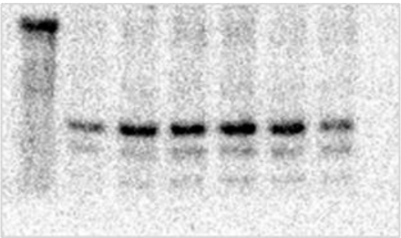
**β-actin**	

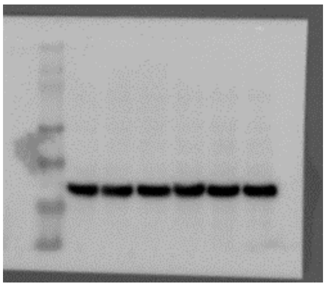	

Figure 7A

p-p38	p38
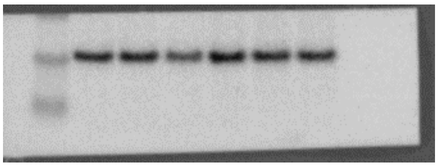	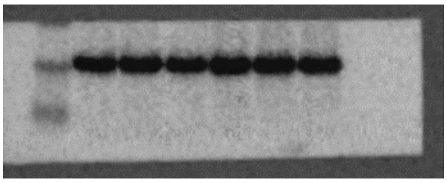
p-ERK	ERK
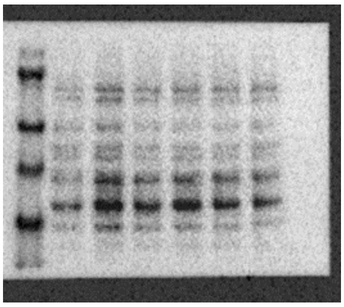	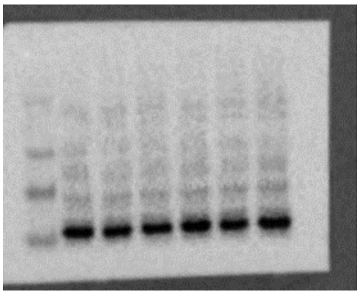
p-JNK	JNK
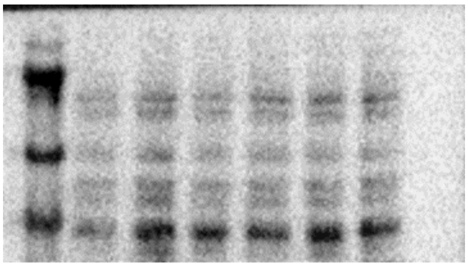	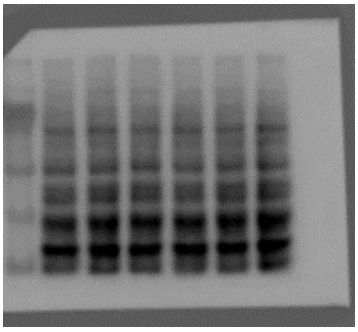
p-STAT1	p-NF-κB
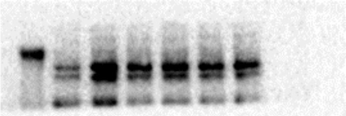	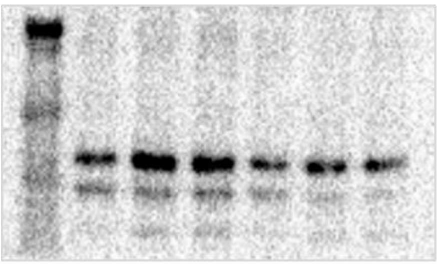
β-actin	

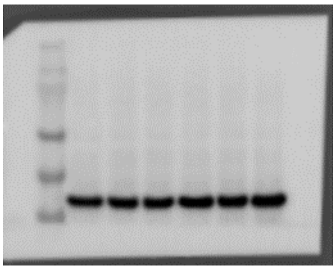

Figure 8D

iNOS	COX2
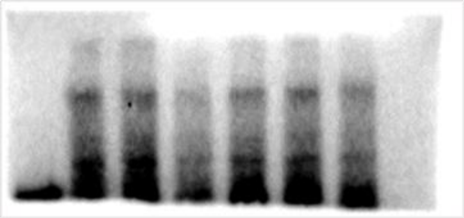	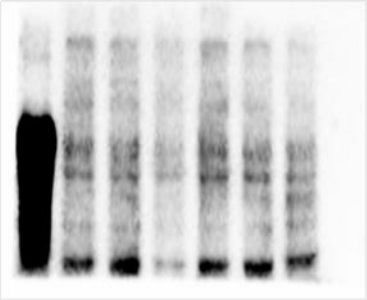
**β-actin**	
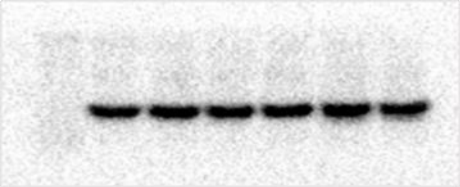	
